# Mediterranean diet and gut microbiota: impact on memory and other cognitive functions: a systematic review

**DOI:** 10.3389/fnmol.2026.1749308

**Published:** 2026-03-10

**Authors:** María Victoria Ibeas-Pérez, Blanca Agüí-Ruiz, Samuel Arias-Sánchez, Isabel Martín-Monzón

**Affiliations:** 1 Department of Experimental Psychology, Faculty of Psychology, Campus Santiago Ramón y Cajal, University of Seville, Seville, Spain; 2 Wellness Neuroscience Group, Department of Experimental Psychology, Faculty of Psychology, Campus Santiago Ramón y Cajal. University of Seville, Seville, Spain

**Keywords:** bacteria, cognition, Mediterranean diet, memory, microbiota, short-chain fatty acids

## Abstract

In recent years, there has been growing interest in the study of gut microbiota and its relationship with multiple diseases, ranging from digestive problems to cognitive disorders. The composition of this microbiota is determined by external and internal factors—such as psychosocial or environmental aspects—and is closely linked to diet, since the foods we consume provide nutrients and establish the conditions of the intestinal environment. The gut–brain axis describes how the intestinal microbial flora and the compounds it produces generate and transmit signals that act on our nervous system and regulate multiple processes in the body. This systematic review aims to explore the impact of the Mediterranean Diet on the composition of the gut microbiota and to analyze its effects on various cognitive conditions, such as memory. Based on a review of 20 articles, we examined how the Mediterranean Diet—characterized by high consumption of olive oil, fruits, vegetables, legumes, and fish—modulates the microbiota in the human gut. The results showed that adherence to the Mediterranean Diet is associated with an increase in beneficial bacteria such as *Faecalibacterium prausnitzii* and *Bifidobacterium*, and with greater production of short-chain fatty acids (SCFAs), especially butyrate. The Mediterranean Diet appears to exert a neuroprotective role in disorders such as mild cognitive impairment, schizophrenia, Parkinson’s disease, and metabolic diseases. This protective function, derived from changes in the gut microbiota, leads to improvements in cognitive function. Overall, the findings underscore the direct relationship between nutrition and mental health and reinforce the value of the Mediterranean Diet as a preventive strategy and a modulator of cognition through the gut–brain axis, promoting brain health across the lifespan.

**Systematic review registration:**

https://www.crd.york.ac.uk/prospero/, identifier CRD420251273990.

## 1 Introduction

The Mediterranean diet, originating in ancient Greece (Radd-Vagenas et al., 2017), is distinguished as a predominantly plant-based eating pattern composed of fruits, vegetables, legumes, nuts, and seeds. It also includes moderate amounts of fish and only small portions of red and processed meat. The primary fat used for cooking and as a dressing is extra-virgin olive oil (EVOO), and together with wine consumed in small quantities during meals these are hallmark features of the diet (Bach-Faig et al., 2011). It is important to emphasize that the Mediterranean diet is not defined by geographic location but by its constituent foods; thus, people living outside the Mediterranean basin may also follow this dietary pattern (Varela, 1994). The Mediterranean diet was introduced as a healthy diet by Ancel Keys through his innovative “Seven Countries Study” in the 1950s, which examined the role of the Mediterranean diet in cardiovascular disease (Keys, 1980). In that work, Keys described the link between the eating practices of certain Mediterranean communities and a lower incidence of cardiovascular disease. In 2006, Scarmeas and colleagues reported an association between adherence to the Mediterranean diet and a decreased risk of developing Alzheimer’s disease (Scarmeas et al., 2006). Compounds found in EVOO have been shown to hold substantial potential for the prevention and treatment of some neurodegenerative diseases, such as Alzheimer’s disease (Alkhalifa et al., 2024), and, more generally, adherence to the Mediterranean diet reduces the risk of chronic disease and increases life expectancy (Estruch et al., 2018). In recent decades, changes in the Mediterranean diet have been observed both in European Mediterranean countries and in regions south of the Mediterranean (Vilarnau et al., 2019). These shifts in food preferences, together with rising food costs and the industrialization of food production, have contributed to a decline in the traditional Mediterranean pattern (Russo et al., 2021), which will likely have repercussions for the health of populations that previously adhered to this diet. In the neurological sphere, a 2024 systematic review suggests that greater adherence to the Mediterranean diet may be associated with better cognitive performance and gastrointestinal symptoms in Parkinson’s disease, accompanied by variations in gut microbial composition (Seelarbokus et al., 2024).

The human intestine harbors a microbial community comprising fungi, bacteria, archaea, and viruses that can reach 10^13^ cells, forming a complex ecosystem known as the gut microbiota, which remains in constant communication with host cells and systems and thereby contributes to our health. Bacteria are the principal constituents of the human microbiota; to date, more than 1,000 different species have been identified, of which each person harbors roughly 500 (Leviatan et al., 2022). These bacteria establish a symbiotic relationship with the host, obtaining nutrients from the gastrointestinal tract and contributing to immunological, structural, and metabolic functions that benefit the host (Bäckhed et al., 2005; O’Hara and Shanahan, 2006). However, the composition of the microbiota is not stable: it can change as a consequence of environmental factors and host-related alterations such as the consumption of different foods, medication use, age, or even excessive personal and environmental hygiene. Among these factors, diet appears to be the most decisive, due to microorganisms’ differing capacities to metabolize specific substrates and to tolerate the intestinal milieu generated by various foods (De Filippo et al., 2017). For example, studies on extra-virgin olive oil report that this oil helps reduce pathogenic bacteria in the intestine and promotes the growth of beneficial bacteria, which is crucial for maintaining microbial balance. Moreover, EVOO consumption increases the production of short-chain fatty acids (SCFAs) synthesized by bacteria, which exert anti-inflammatory effects and can also influence host gene expression (Millman et al., 2021). Contemporary diets rich in proteins, sugars, and fats and including non-food chemicals such as preservatives, pesticides, additives, and emulsifiers provoke shifts in microbiota composition and, consequently, in host–microbe relationships (Sonnenburg and Sonnenburg, 2019). These alterations can contribute to pathologies such as inflammatory bowel disease, cardiovascular disease, diabetes, obesity, allergies, and metabolic syndromes, among others (Dapa and Xavier, 2024). The microbiota has also been shown to affect drug treatments, enhancing, inhibiting, or modifying their activity (Manrique et al., 2024). All of this has fueled scientific interest in the microbiota and in the mechanisms underlying host–microbe interactions, as well as in the role of diet in reshaping the microbiota to benefit health. At present, numerous studies analyze the microbiota of humans with diverse pathologies—from intestinal to neurological conditions—comparing findings with healthy individuals to elucidate key relationships; many other studies likewise assess the impact of diet on the microbiota. Traditional culture-based techniques have been used to study the microbiota; however, many bacteria that compose it cannot be grown outside the intestine or are present at very low abundance, preventing their isolation and identification. To address this issue, new culture methods such as microfluidics are being developed; nevertheless, identification is currently carried out primarily using molecular methods. Total DNA is extracted from fecal samples from the individuals under study and, through sequencing of the 16S rRNA gene or shotgun metagenomic sequencing of the extracted DNA, the bacterial species present and their relative abundances in the sample are determined (Xu et al., 2024).

For years it has been recognized that there is a connection between the gut and the brain known as the gut–brain axis, which constitutes a bidirectional communication route essential for maintaining homeostasis. This axis is composed of the microbiota, the enteric nervous system, the autonomic nervous system, the neuroendocrine system, the neuroimmune system, and the central nervous system (Barrio et al., 2022; Felice et al., 2016). Neurochemical signaling and the vagus nerve are among the mechanisms that transmit signals along this axis (O’Mahony et al., 2015; O’Riordan et al., 2025). As expected, dysfunction of this complex system has important pathophysiological consequences, and numerous brain disorders have been linked to alterations in microbiota composition, with dysbiosis and disease-related molecular changes detected in affected patients (Mayer, 2011). One communication mechanism within the gut–brain axis is the immune system, both through the production of immunomodulators by the resident microflora and via direct interactions between these bacteria and intestinal immune cells, given that the gut constitutes a major immune niche (Shekarabi et al., 2024). Among the major microbiota-derived mediators that modulate immunity and neuro-immune signaling are SCFAs, secondary bile acids, neuromodulators/neurotransmitters (e.g., GABA, serotonin/5-HT and their precursors), and choline-derived metabolites (TMA/TMAO) (Connell et al., 2022; Park et al., 2025). Of these, SCFAs are among the most studied: they are generated when gut microbes ferment dietary fibers that escape human digestion and act on immune, endocrine, and neuronal cells (Dalile et al., 2019). Adhering to a healthy diet can increase microbial diversity in the gut and enhance the production of these fatty acids and other bioactive compounds. In this regard, a reduction in the relative abundance of SCFA-producing bacterial genera has been linked to cognitive pathologies (Ribeiro et al., 2022). Regarding neuromodulators, some components of the microbiota can produce GABA, the principal inhibitory neurotransmitter in the nervous system (Strandwitz et al., 2019). In parallel, direct interactions between intestinal bacteria and gut immune cells trigger the release of cytokines and chemokines that enter the circulatory and lymphatic systems and influence immune signaling throughout the body, including the brain (Erny et al., 2015). Recent updates underscore the immune system as a central pathway of the microbiota–gut–brain axis: the microbiota “educates” innate and adaptive immune responses, shapes microglial function and neuroinflammation, and opens therapeutic opportunities in neurological disorders (O’Riordan et al., 2025). Another signaling route within the axis operates through intrinsic enteric neurons, which relay signals to the sympathetic ganglia, and through the vagus nerve, which expresses a variety of receptors along the gut–vagus–brain pathway and informs complex behaviors such as food preference, motivation, and reward (Shekarabi et al., 2024). Taken together with current clinical evidence, these mechanisms help explain why adherence to the Mediterranean diet may translate into more favorable microbial profiles and the cognitive benefits observed in recent cohorts and systematic reviews (Seelarbokus et al., 2024; Tessier et al., 2024). Given these considerations, the objective of this systematic review is to examine the relationship between adherence to the Mediterranean diet and gut microbiota composition within the context of the gut–brain axis, and to evaluate its impact on memory and other cognitive functions, with particular attention to microbiome-related changes.

## 2 Methods

### 2.1 Search strategy

**FIGURE 1:**
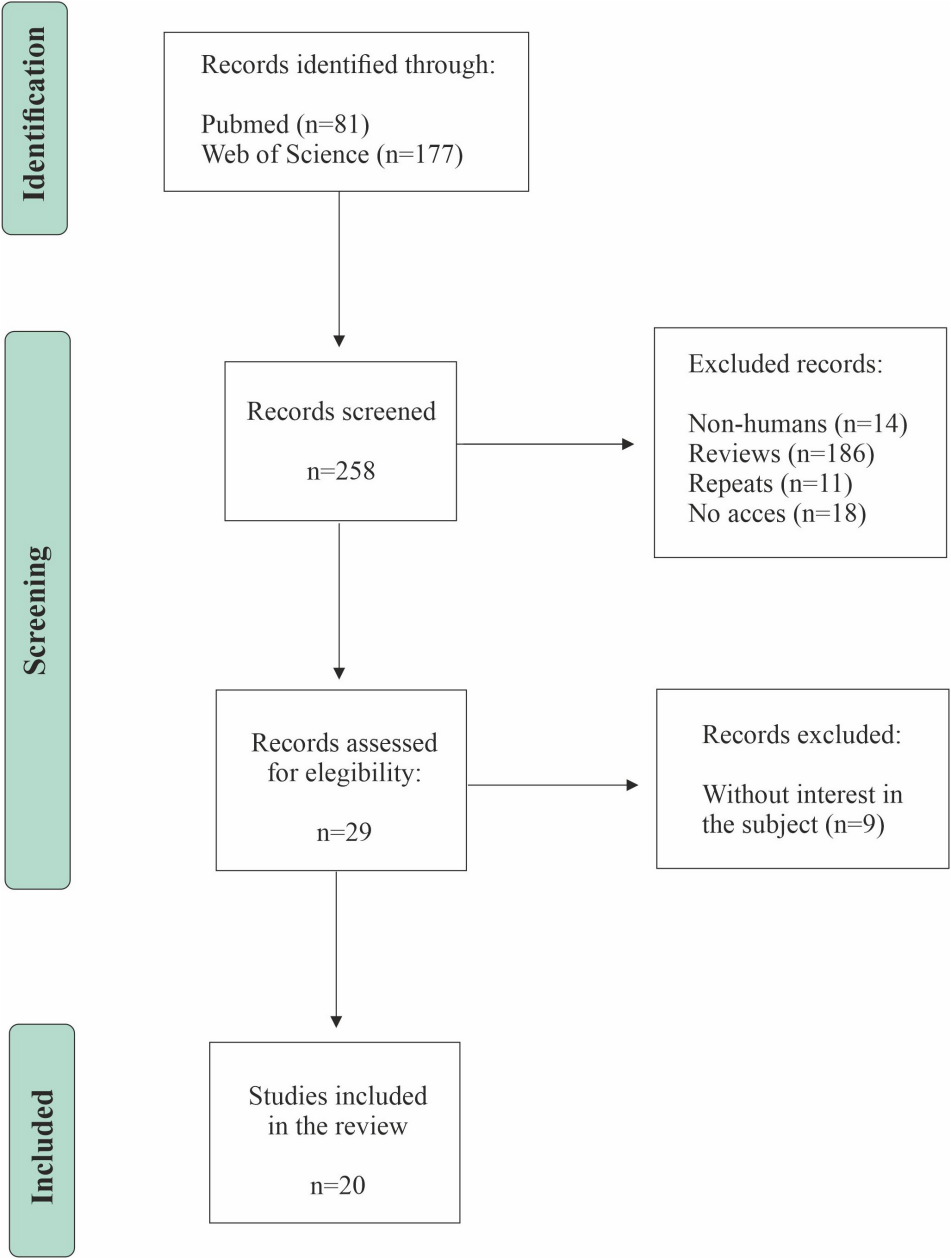
PRISMA flow diagram of the study selection.

A systematic review of the scientific literature was conducted following the PRISMA (Preferred Reporting Items for Systematic Reviews and Meta-Analyses) guidelines (Liberti et al., 2009; Moher et al., 2009) to identify the effect of the Mediterranean diet on memory and other cognitive processes, the microbiota population influenced by it, and how these bacteria influence the memory system. Details of the protocol for this systematic review were registered in the PROSPERO database (CRD420251273990). The search strategy was built around the three core concepts defining our research question: “Mediterranean diet,” “Memory,” and “Microbiota.” These key terms were combined using the Boolean operator “AND” to ensure precise retrieval of studies investigating their intersection. The specific syntax was adapted for each database. The study selection process followed four distinct stages: (1) Identification: Records obtained from databases were imported into reference management software, and duplicates were removed. (2) Screening: Titles and abstracts of all unique records were screened against pre-defined eligibility criteria. (3) Eligibility: The full texts of potentially relevant studies were retrieved and assessed in detail for inclusion. (4) Inclusion: Studies meeting all criteria were finally included in the qualitative synthesis. This process was performed independently by two reviewers, with any discrepancies resolved through discussion or consultation with a third reviewer. The complete flow of information through these stages, including the number of records at each point, is presented in the PRISMA flow diagram (Figure 1).

### 2.2 Study selection

Specific exclusion criteria were established to focus the search on relevant articles. First, systematic reviews were excluded, as well as studies based on research using animal models, including only empirical studies. This was done to ensure that the results could be directly applied to the human population, which is crucial both for clinical use and for advancing the understanding of neuroscience in relation to humans. Likewise, studies that did not explicitly focus on changes in the microbiota, memory, or the gut-brain axis were discarded, in order to accurately address the research objective proposed in this paper. Finally, articles published before 2014 were excluded to ensure that the selected studies reflected the current level of knowledge in the field of neuroscience. With regard to publication characteristics, no conference abstracts, preprints, or non-English publications met the inclusion criteria at the title and abstract screening stage; therefore, no studies were excluded on the basis of publication type or language.

A total of 258 articles were located (81 from PubMed and 188 from Web of Science). After eliminating duplicates, an initial screening was performed, and 29 articles were selected for full reading. During this second phase, relevant methodological aspects were verified, such as sample characteristics, cognitive tools used, and microbiota analysis techniques. Finally, new studies were excluded for not meeting the eligibility criteria, leaving a total of 20 studies included in the review (Table 1). The selection process is summarized in Figure 1.

**TABLE 1:** Articles included in the systematic review.

Code	First author and year	Journal	Cites	Mediterranean diet adherence measure	Quality assessment	
					Tool	Score/Judgment
1	(Cupisti et al., 2017)	Nutrients	53	NA	NA	NA
2	(Nagpal et al., 2020)	PLOS ONE	46	NR	NOS	5/9
3	(Godos et al., 2021)	Nutrients	36	MEDI-LITE	NOS	6/9
4	(Berding et al., 2023)	Microbiome	63	ModiMedDiet	RoB 2	Some concerns
5	(Haskell-Ramsay et al., 2022)	Psychopharmacology	4	NA	RoB 2	Some concerns
6	(Pellegrini et al., 2020)	Food Chemistry	48	IMI	RoB 2	High risk/Some concerns
7	(Domínguez-López et al., 2024)	Frontiers in Nutrition	0	MEDAS-14	NOS	6/9
8	(Domínguez-López et al., 2023)	Molecular Nutrition and Food Research	1	MEDAS-17	NOS	6/9
9	(Kowalski et al., 2023)	Clinical Nutrition	8	aMED	NOS	7/9
10	(McLeod, 2021)	Nutrients	0	aMED	RoB 2	Some concerns
11	(Paknahad et al., 2020)	Nutrients	62	NR	RoB 2	Some concerns
12	(van Soest et al., 2020)	Nutrients	51	NR	NOS	6/9
13	(Santoro et al., 2014)	Mechanisms of Ageing and Development	67	NR	NA	NA
14	(Cardelo et al., 2022)	Frontiers in Nutrition	3	MEDAS-14	RoB 2	Some concerns
15	(Kamer et al., 2023)	Gut Microbes	4	NR	RoB 2	Low risk/Some concerns
16	(McLeod et al., 2023)	Nutrients	1	MDS	RoB 2	Some concerns
17	(O’Mahony et al., 2023)	Frontiers in Nutrition	1	NA	NA	NA
18	(Nagpal et al., 2020)	Trends in Endocrinology and Metabolism	5	NA	RoB 2	Some concerns
19	(Galié et al., 2021)	Journal of Clinical Medicine	5	MEDAS-17	RoB 2	Some concerns
20	(Choo et al., 2023)	Nutrients	5	MEDAS-14; MDS	RoB 2	Some concerns

^1^aMED, Alternate Mediterranean Diet score; IMI: Italian Mediterranean Index; MDS, Mediterranean Diet Score; MEDAS-14, Mediterranean Diet Adherence Screener, 14-item; MEDAS-17, Mediterranean Diet Adherence Screener, 17-item; ModiMedDiet: Modified Mediterranean Diet Score; NA, Not applicable; NOS, Newcastle–Ottawa Scale; NR, Not reported.

^2^Observational studies were appraised with the Newcastle–Ottawa Scale (NOS; 0–9 stars). Randomized controlled trials were assessed with RoB 2 (overall judgment: low risk/some concerns/high risk). Non-primary articles (e.g., protocols/reviews) were not assessed (NA).

### 2.3 Quality assessment and risk of bias

The methodological quality of the included studies was assessed according to study design. Observational studies were evaluated using the Newcastle-Ottawa Scale, while randomized controlled trials were assessed using the Cochrane Risk of Bias tool. Quality scores were considered in the interpretation of findings to account for methodological heterogeneity across studies.

## 3 Results

Tables 2–4 summarize the studies reviewed in the context of gut microbiota, the Mediterranean diet, and cognitive processes. The results presented in Table 2 show that, in both observational studies and controlled interventions, adherence to the Mediterranean diet is associated with beneficial changes in the gut microbiota and improved cognitive function. These effects are especially notable in populations with mild cognitive impairment, obesity, or aging. The Mediterranean diet favors the increase of bacteria such as *Faecalibacterium prausnitzii*, *Akkermansia muciniphila*, *Bifidobacterium*, and *Lactobacillus*, which increase the production of short-chain fatty acids (butyrate, propionate, and acetate) with anti-inflammatory and neuroprotective effects. The presence of these metabolites has been linked to improved performance in verbal memory, executive functions, and frontal cognition (Domínguez-López et al., 2024; van Soest et al., 2020). Furthermore, bioactive components of the Mediterranean diet, such as polyphenols present in fruits, vegetables, nuts, and extra virgin olive oil, could enhance these benefits by modulating the gut microbiota (Godos et al., 2021).

**TABLE 2:** Mediterranean diet, microbiota and cognition.

Code	*n*	Age	Procedure	Pathology	Control/comparison group	Main results
16	185	70	MD intervention with weight loss	Obesity and advanced age	Comparison MD, MD + weight loss, and habitual diet	Improved memory; ↑ SCFAs
11	80	58.9	Controlled MD trial	Parkinson	Control group with standard diet	Cognitive improvement (verbal memory)
14	47	60	MD + probiotics intervention	Mild cognitive impairment	Comparison with group without probiotics	Improved executive functions and memory; ↑ *Bifidobacterium*
7	200	66	Observational	–	–	SCFAs and phenols associated with better frontal cognition
12	252	72	MD Intervention	–	Control group	↑ *Faecalibacterium* cognitive improvement
6	342	67	Clinical trial with MD	Mild cognitive impairment	Control group	Microbiota changes associated with improved verbal memory
3	2044	34	Observational	-	-	Phenols correlated with better cognitive status

In alphabetical order: ↑, increase; MD, Mediterranean diet; SCFAs, Short Chain Fatty Acids.

**TABLE 3:** Mediterranean diet and microbiota.

Code	n	Age	Procedure	Pathology	Control/comparison group	Main results
19	38	42.5	MD Intervention	Cardiometabolic risk factors	Control group	↑ *Lactobacillus* ↑ *Bifidobacterium*
20	34	60	MD + nuts intervention	-	Comparison with low-fat diet	↑ Microbial diversity;, *Akkermansia muciniphila*
1	–	–	Dietary intervention	Overweight	Standard diet	Microbiota composition changes
13	1250	72	Dietary intervention	–	Control group	Improved microbiota composition
15	82	52	Intervención dieta	Overweight	Standard diet	↑ SCFAs

In alphabetical order: ↑: increase; MD, Mediterranean diet; SCFAs, Short Chain Fatty Acids.

**TABLE 4:** Microbiota and cognition.

Code	n	Age	Procedure	Pathology	Comparison/control group	Main results
9	155	41.5	Observational	SQZ	Comparison of microbial profiles	↓ SCFAs associated with poorer cognitive performance.
8	400	67.5	Observational	Overweight	-	Phenols correlated with better frontal lobe function.
5	79	33.5	Nuts intervention	-	Placebo	Microbiota changes; slight improvement in working memory.
4	45	38.5	Probiotic intervention	Autism	Control group	Improved attention and memory.
17	47	64.5	Red fruits intervention	-	Control group	Microbiota changes; mild memory improvement.
18	17	64.6	Dietary intervention	-	Control group	Microbiota changes associated with better cognitive function.
2	15	39.2	Fermented diet intervention	-	Control group	Improved microbiota and verbal memory.
10	185	70	Probiotic intervention	Obesity	Placebo group	Improvement in working memory and sustained attention.

In alphabetical order: ↓: decrease; SCFAs, Short Chain Fatty Acids; SQZ, schizophrenia.

Table 3 includes studies in which DM modulates the microbiota without directly assessing cognitive functions. An increase in *Butyricicoccus* and a reduction in *Colinsella* and *Veillonella* observed. These changes reflect an anti-inflammatory profile that could explain the indirect cognitive benefits reported (Choo et al., 2023; Galié et al., 2021).

Table 4 details that an unbalanced microbiota with low SCFA production and lower bacterial diversity is associated with worse cognitive performance and higher risk of decline. In contrast, interventions involving MD, probiotics or polyphenols have been shown to improve cognition (Berding et al., 2023; Nagpal et al., 2020).

The assessment methods used across studies included neuropsychological scales such as the Mini-Mental State Examination (MMSE), the Trail Making Test (TMT), and the Montreal Cognitive Assessment (MoCA), with more consistent results being in long-term interventions combined with probiotics or polyphenolic compounds. Overall, the evidence supports that MD acts as a key modulator of the gut-brain axis, promoting an anti-inflammatory microbiota that contributes to cognitive health in different populations (Table 5).

**TABLE 5:** Summary of findings according to the analyzed.

Area of impact	Main findings	Relevant studies
Gut microbiota	Increased abundance of beneficial bacteria such as *Faecalibacterium*, *Bifidobacterium*, *Akkermansia*, *Lactobacillus.*	(van Soest et al., 2020; Choo et al., 2023)
Microbial metabolites	↑ SCFAs –butyrate, propionate, acetate — involved in inflammation regulation and synaptic plasticity.	(Domínguez-López et al., 2024; Joseph et al., 2017)
Cognitive function	Improvement in verbal memory, attention, and executive functions.	(Paknahad et al., 2020; Berding et al., 2023)
Pathology	Positive effects observed in mild cognitive impairment, Parkinson’s disease, autism, cardiovascular disorders, and schizophrenia.	(Pellegrini et al., 2020; Kowalski et al., 2023)
Type of intervention	Mediterranean diet alone or combined with probiotics/nuts improves microbial composition and cognition.	(Galié et al., 2021; Godos et al., 2021)

In alphabetical order: ↑: increase; SCFAs, Short Chain Fatty Acids.

## 4 Discussion

In the present work, a systematic review of studies published over the past decade was conducted to examine the influence of the Mediterranean diet on memory and other cognitive processes through its effects on the gut microbiota. The evidence indicates that adherence to this dietary pattern is associated with favorable modifications in gut microbial composition, characterized by an increase in beneficial bacteria with anti-inflammatory and neuroprotective properties. These microbiota-related changes appear to contribute to improved cognitive performance and a lower risk of cognitive decline across diverse populations.

When we examined the gut–brain axis, we observed associations between specific bacterial populations and the metabolites they produce and the presence or absence of cognitive pathologies. Finally, analysis of the Mediterranean diet’s impact on cognitive processes indicates that people adhering to this diet are less likely to experience such disorders. Overall, the evidence suggests that a greater abundance of beneficial bacteria, fostered by adherence to the Mediterranean diet, is associated with increased production of short-chain fatty acids. These microbial changes appear to play a key role in neuroprotection and in improving cognitive performance across different populations.

### 4.1 Mediterranean diet and the microbiota

The stable presence of microorganisms in the intestine has been recognized for over a century, but it was not until the late twentieth century thanks to the development of molecular identification techniques that this could be demonstrated empirically and the enormous number of distinct groups present, as well as the variability in their proportions, could be determined (Almansour et al., 2025). Much of the interindividual variation in microbiota composition is determined by diet. The studies reviewed here on the interaction between the Mediterranean diet and the gut microbiota (Table 3) show benefits in microbial composition, including increases in Butyricicoccus and decreases in Collinsella and Veillonella, changes linked to an anti-inflammatory profile that support the idea that the Mediterranean diet can modulate the intestinal milieu (Galié et al., 2021). In addition, several studies (Cupisti et al., 2017; Kamer et al., 2023) highlight specific benefits in individuals with overweight or obesity, in whom Mediterranean-diet interventions led to increases in *Lactobacillus* and *Bifidobacterium*. Taken together, these findings suggest that the Mediterranean diet may exert a positive impact both in healthy populations and in those with excess weight or metabolic risk.

### 4.2 Microbiota and cognitive processes

Consistent with broader theoretical models, as early as the 4th century BCE, Hippocrates posited a relationship between the gut and health summarized in phrases such as “bad digestion is the root of all evil” and “death sits in the bowels” (Sekirov et al., 2010). The analysis of the relationship between the microbiota and cognition conducted in this review (Table 4) shows that poorer cognitive performance is associated with dysbiosis characterized by low production of SCFAs. This is observed, for example, in patients with schizophrenia and overweight (Kowalski et al., 2023; Domínguez-López et al., 2023). Conversely, the consumption of fermented foods, berries, nuts, and probiotic supplements improves working memory and attention across different populations. Notably, some studies implementing interventions with *Lactobacillus plantarum* in individuals with autism reported positive effects on attention and memory (Berding and Cryan, 2022). These results, among the studies included in our systematic review (Nagpal et al., 2020); indicate that the gut microbiota plays a mediating role in cognitive functions via systemic inflammation, blood–brain barrier integrity, and the synthesis of neuroactive metabolites such as butyrate, and align with the broader hypothesis of the gut-brain axis modulation (Barrio et al., 2022; Joseph et al., 2017).

### 4.3 Mediterranean diet and cognitive processes

The consumption of diverse foods—both natural and processed—has been associated with beneficial health effects across different populations. For example, the intake of fermented foods, rich in pre- and probiotics, has been linked to effects on the immune system and metabolic functions, as proposed in mechanistic models (Mota de Carvalho et al., 2018). Beyond these and other well-established health effects, adherence to the Mediterranean diet has been associated with a reduced risk of cardiovascular disease (Nestel and Mori, 2022). In recent years, growing evidence has also correlated specific dietary patterns with mental health. Notably, balanced diets complemented with nutritional supplements—or the MD itself—may contribute to brain development and improve mental health outcomes (Das et al., 2025).

Based on the findings from the cohort studies included in our systematic review, analyses of the MD’s role in cognitive processes indicate that, in conditions such as Parkinson’s disease and mild cognitive impairment (MCI), interventions demonstrate significant improvements in verbal memory and executive functions. In Parkinson’s disease, a specific improvement in verbal memory has been reported (Paknahad et al., 2020), whereas in MCI, interventions combining the MD with probiotics improved not only attention but also executive function (Cardelo et al., 2022). Benefits have likewise been observed in community-dwelling older adults, where higher levels of short-chain fatty acids (SCFAs) correlate with better performance on memory tasks and frontal cognition (Domínguez-López et al., 2024; van Soest et al., 2020).

### 4.4 Mediterranean diet, microbiota, and cognitive processes

#### 4.4.1 Microbiota-mediated mechanisms linking the Mediterranean diet to cognitive function

Recent studies have shown that the beneficial effects of certain diets are achieved through modulation of the gut microbiota, thereby linking diet with the intestinal microbiota and overall health (Muigano et al., 2025) and, more specifically, with mental health (Morse and Garcia, 2025; Patil and Mehdi, 2025). Based on the studies selected for this review, the results indicate a role for the Mediterranean diet as a neuroprotective factor for the central nervous system (CNS) and highlight its influence on the bacterial populations of the gut microbiota which, through the production of short-chain fatty acids, modulate cognitive processes such as memory. In particular, propionate, butyrate, and acetate are essential for modulating brain function because, after crossing the blood–brain barrier, they reduce inflammation and enhance synaptic plasticity. This anti-inflammatory mechanism could underlie the reduced risk of cognitive decline observed in different populations following microbiota changes induced by diet specifically, the Mediterranean pattern. This suggests that gut microbiota is involved not only in metabolic health but also in mental and cognitive health, linking bacterial community structure with functions such as memory and attention.

#### 4.4.2 Evidence from dietary interventions, probiotics, and cognitive outcomes

In the NU-AGE project (Ghosh et al., 2020), a 12-month Mediterranean-style dietary intervention in older adults decreased systemic inflammation, improved microbiota composition, and was associated with better cognitive function. Likewise, greater adherence to this diet among younger adults correlates with better cognitive performance, supporting a protective effect across the lifespan (Godos et al., 2021). These benefits appear to be driven by an increase in bacteria with anti-inflammatory properties—such as *Faecalibacterium prausnitzii*, *Akkermansia muciniphila*, *Bifidobacterium*, and *Lactobacillus*—which produce SCFAs, especially butyrate. This metabolite participates in epigenetic processes that regulate gene expression in the nervous system, promoting neurogenesis, synaptic plasticity, and modulation of immune responses. Beyond microbial actions, the positive effects also reflect the MD’s composition—rich in neuroprotective nutrients such as polyphenols, antioxidant vitamins, the fatty acids in olive oil, and omega-3s from fish. Together, these factors may directly or indirectly contribute to improved cognitive functions, arising from the interaction between dietary components and microbial effects (Nagpal et al., 2020).

In the same line of research, combining the Mediterranean diet with probiotic supplementation appears to potentiate positive effects on cognition, especially in individuals with neurodevelopmental disorders such as autism (Berding et al., 2023). The cognitive impact of specific strains has also been demonstrated in other articles besides those selected in this review: *Bifidobacterium* significantly improved immediate memory and attention in healthy older adults after 8 weeks of supplementation (Shi et al., 2022); *Lactiplantibacillus* exerted a protective effect on memory function in older adults after a 12-week intervention (Sakurai et al., 2022); and *Lactobacillus rhamnosus* was associated with better cognitive performance in middle-aged and older adults with cognitive impairment after 3 months (Sanborn et al., 2020). These findings open potential avenues for personalized interventions tailored to an individual’s microbial profile, with the goal of mitigating the impact of interindividual variability on cognitive function.

#### 4.4.3 Interindividual variability and complexity of diet–microbiota–cognition interactions

Although most studies report favorable outcomes, microbiota changes induced by the Mediterranean diet have also been documented without significant cognitive improvement. For instance, Cupisti et al. (2017) reported changes in gut microbiota composition without concomitant improvements in cognitive outcomes. This discrepancy may be explained by factors such as the short duration of the intervention, baseline cognitive status, or the sensitivity of the cognitive assessment tools employed, suggesting that microbiota modulation alone may not be sufficient to elicit measurable cognitive effects. Similarly, some studies (Nagpal et al., 2020; Schneider et al., 2024) suggest that the cognitive impact associated with the MD may depend on each subject’s baseline microbiota. This would imply that the Mediterranean diet acts as a modulator, adding another layer of complexity to the bidirectional gut–brain axis.

Summarizing the studies reviewed (Table 5), an association can be established between SCFA production and improvements in synaptic plasticity and inflammatory regulation, supporting a diet-driven modulatory role on the gut–brain axis. Moreover, combining the MD with probiotics or polyphenol-rich foods appears to enhance these effects. Building on these findings, future research may focus on designing personalized interventions informed by baseline microbiota profiles. Still, the studies reviewed exhibit limitations inherent to a nascent field with a relatively limited evidence base. Most studies focus on older adults, introducing potential bias related to age-associated physiological changes. Accordingly, future studies should examine the impact of the Mediterranean diet among younger populations to improve the generalizability of findings. Furthermore, sample sizes in many trials remain modest—rarely surpassing two hundred participants—thus constraining statistical power. Inconsistencies in intervention duration further complicate interpretation: some trials span 12 months, whereas others assess outcomes after as little as 4 weeks, a timeframe unlikely to reflect lasting cognitive effects. Furthermore, there is a lack of standardization in assessment tools for both microbiota analysis and cognitive evaluation. While several studies employ well-established instruments such as the MMSE or MoCA (Pellegrini et al., 2020; van Soest et al., 2020; Santoro et al., 2014), others rely on non-standardized or condition-specific batteries (Berding et al., 2023; Paknahad et al., 2020; Godos et al., 2021), complicating the direct comparison of results.

In summary, considering these limitations and the novelty of this research area, future studies should integrate psychosocial variables—such as stress and social isolation—and examine their relationship with the gut–brain axis. Research addressing sex and gender differences is also warranted, given the influence of sex hormones on microbiota composition. Moreover, greater population diversity, including studies involving children, adolescents, and young adults, along with long-term designs, is essential to evaluate the sustained cognitive benefits of Mediterranean-diet interventions.

### 4.5 Strengths and limitations of the study

This systematic review presents several methodological strengths. First, it adheres to PRISMA guidelines, ensuring a transparent and reproducible study-selection process. The use of two major scientific databases (PubMed and Web of Science) and clearly predefined inclusion and exclusion criteria enhances the rigor and comprehensiveness of the literature search. By restricting the review to human studies and excluding animal models and secondary reviews, the analysis is firmly grounded in clinically relevant evidence, thereby increasing its translational value. Additionally, the integration of findings across three domains (Mediterranean diet, gut microbiota composition, and cognitive function) offers a multidimensional perspective that aligns with current models of the microbiota–gut–brain axis. The synthesis also highlights convergent mechanisms, such as short-chain fatty acid production and neuroinflammatory modulation, thus contributing to a mechanistic understanding of diet–microbiota–cognition relationships. Finally, the review spans a decade of research, incorporating recent developments in metagenomic sequencing and neurocognitive assessment, which strengthens the contemporaneity of the conclusions.

However, important limitations must also be acknowledged. The evidence base remains heterogeneous, with considerable variability in sample sizes, intervention duration, and population characteristics, which restricts comparability across studies. While this review provides a focused synthesis of the interrelationship between the Mediterranean diet, memory, and microbiota, certain methodological choices warrant consideration. The search strategy was deliberately precise, employing the three key terms (“Mediterranean diet,” “Memory,” “Microbiota”) connected by “AND” to directly address our specific research question. This ensured high relevance and manageability of the retrieved corpus. However, this precision may also constitute a limitation, as it potentially excludes relevant studies that use synonymous or adjacent terminology for any of these core concepts (e.g., “healthy diet,” “cognitive function,” “gut microbiome”). Future reviews aiming for broader coverage could consider employing expanded search strings with controlled vocabulary and a wider array of synonyms. An important limitation across the included studies is the substantial methodological heterogeneity in microbiota analysis. Differences were observed in sequencing platforms, biological sample types, and bioinformatics and statistical pipelines used for data processing and interpretation. Such variability hampers direct comparison of microbiota-related findings across studies and may partly explain inconsistencies in reported associations. These methodological differences should be carefully standardization in future research. Microbiota analysis methods and cognitive assessment tools are likewise inconsistent, complicating the integration of results. Most studies involve older adults or clinical populations, limiting generalizability, while key confounders such as medication use, lifestyle factors, and baseline microbial profiles are not uniformly controlled. These considerations highlight the need for more standardized, longitudinal, and methodologically robust studies to strengthen the reliability of future conclusions.

A key methodological aspect of this review is the heterogeneity in dietary assessment tools and the rigor of the quality appraisal. Regarding diet, about half of the included studies (11/20; ∼55%) assessed dietary intake using Food Frequency Questionnaires (FFQs), often alongside classic indices such as the 14-item MEDAS. The remaining studies mainly relied on other self-reported approaches, including 24-h dietary recalls and/or short dietary records. However, recent investigations from the PREDIMED-Plus project (Domínguez-López et al., 2023, 2024) have introduced greater precision through the use of objective nutritional biomarkers, namely urinary phenolic metabolites, which can complement questionnaire-based measures and help mitigate recall bias.

To assess the quality of evidence, specific tools were selected according to the epidemiological design of the included studies. Observational studies were evaluated using the Newcastle–Ottawa Scale (NOS), showing overall medium quality (range 5–7/9), reflecting generally acceptable participant selection and some control of confounding, although limited by the predominance of cross-sectional designs. Randomized controlled trials (RCTs) were analyzed using the Cochrane Risk of Bias 2 (RoB 2) tool; most trials showed a low risk of bias or some concerns, mainly due to the difficulty of blinding dietary interventions, an inherent challenge in nutrition studies. Overall, the combined use of NOS and RoB 2 ensures that the internal validity of each finding has been judged against the appropriate standard for its design.

## 5 Conclusion

In this systematic review, the available evidence suggests that modulation of the gut microbiota through dietary patterns rich in fiber, antioxidants, and anti-inflammatory compounds—such as the Mediterranean diet- may represent a promising mechanism for preventing and mitigating neurodegenerative diseases, as well as a key strategy to promote brain health.

Across the studies analyzed, adherence to this dietary pattern is consistently associated with a greater abundance of beneficial bacteria such as *Faecalibacterium prausnitzii* and *Bifidobacterium*, along with increased production of short-chain fatty acids. These metabolites, particularly butyrate, are involved in processes such as synaptic plasticity and the regulation of inflammation, which may underlie the observed improvements in memory, attention, and other executive functions across diverse populations. Nonetheless, ongoing technological advancements are required to enable more granular microbiome analyses and generate higher-precision data on how the Mediterranean diet modulates microbial ecology and brain-health biomarkers. This need is underscored by the considerable heterogeneity observed across current studies in terms of sample sizes, intervention duration, and methodological approaches, which limits comparability and highlights the importance of developing more standardized and robust research frameworks.

On the basis of these findings, the Mediterranean diet can be regarded not only as a healthy eating pattern but also as an intervention strategy with the potential to enhance multiple cognitive domains—including memory, attention, and executive functions—particularly when complemented by selected probiotics. This integrative approach opens new avenues for personalized and preventive programs tailored to diverse population groups and specific risk profiles.

In summary, this review underscores the relevance of nutrition in brain function and positions the Mediterranean diet as a potentially beneficial dietary pattern in the prevention of neurodegenerative diseases and the promotion of cognitive performance and brain health across the lifespan.

This research did not receive any specific grant from funding agencies in the public, commercial, or not-for-profit sectors.

## Data Availability

The original contributions presented in this study are included in this article/supplementary material, further inquiries can be directed to the corresponding author.
